# Large Doses of Quinine in Epilepsy

**Published:** 1844-10-19

**Authors:** 


#### LARGE DOSES OF QUININE IN EPILEPSY.

M. Taroni mentions in the Gazetta Medica di Milano the case of a young woman who was the subject of epilepsy from fright. Failing other measures, and the disease manifesting marked periodicity, M. Taroni exhibited quinine in large doses, beginning with twenty grains daily, and gradually raising the quantity to forty grains, which were given daily for six days, after which the dose was gradually diminished. During all this while the cure was progressive, and was finally accomplished.—*Ibid.*

